# In Vivo Bacteriophages’ Application for the Prevention and Therapy of Aquaculture Animals–Chosen Aspects

**DOI:** 10.3390/ani12101233

**Published:** 2022-05-10

**Authors:** Patrycja Schulz, Joanna Pajdak-Czaus, Andrzej Krzysztof Siwicki

**Affiliations:** 1 Department of Ichthyopathology and Fish Health Prevention, S. Sakowicz Inland Fisheries Institute, Główna 48, 05-500 Żabieniec, Poland; 2 Department of Epizootiology, Faculty of Veterinary Medicine, University of Warmia and Mazury in Olsztyn, Oczapowskiego 13, 10-719 Olsztyn, Poland; joanna.pajdak@uwm.edu.pl; 3 Department of Microbiology and Clinical Immunology, Faculty of Veterinary Medicine, University of Warmia and Mazury in Olsztyn, Oczapowskiego 13, 10-719 Olsztyn, Poland; siwicki@uwm.edu.pl

**Keywords:** bacteriophages, fish, bacterial diseases, prevention, therapy

## Abstract

**Simple Summary:**

The world’s population is projected to reach 10 billion by 2050. To meet the nutritional needs of this growing population, animal production must double by 2050. The production of fish and other aquatic animals is growing rapidly, but with an intensification of farming, the risk of infectious diseases is increasing, including bacterial diseases. In recent years, antibiotics and chemotherapeutic agents that can be used in aquaculture have been evidenced as no longer effective, resulting in a lack of effective treatment options and leading to higher animal mortality and economic losses for the farm. For this reason, new prevention and treatment options are being sought. One such method is the use of bacteriophages. These are viruses that attack bacteria, consequently destroying them. This is not a new idea, as the first scientific reports on the use of bacteriophages on animals in aquaculture were published 40 years ago but were abandoned after the invention of antibiotics. Now, they are rapidly gaining renewed interest. This paper summarizes the results of using bacteriophages in various aquaculture animals for the prevention and control of bacterial pathogens.

**Abstract:**

To meet the nutritional requirements of our growing population, animal production must double by 2050, and due to the exhaustion of environmental capacity, any growth will have to come from aquaculture. Aquaculture is currently undergoing a dynamic development, but the intensification of production increases the risk of bacterial diseases. In recent years, there has been a drastic development in the resistance of pathogenic bacteria to antibiotics and chemotherapeutic agents approved for use, which has also taken place in aquaculture. Consequently, animal mortality and economic losses in livestock have increased. The use of drugs in closed systems is an additional challenge as it can damage biological filters. For this reason, there has been a growing interest in natural methods of combating pathogens. One of the methods is the use of bacteriophages both for prophylactic purposes and therapy. This work summarizes the diverse results of the in vivo application of bacteriophages for the prevention and control of bacterial pathogens in aquatic animals to provide a reference for further research on bacteriophages in aquaculture and to compare major achievements in the field.

## 1. Introduction

**Figure 1:**
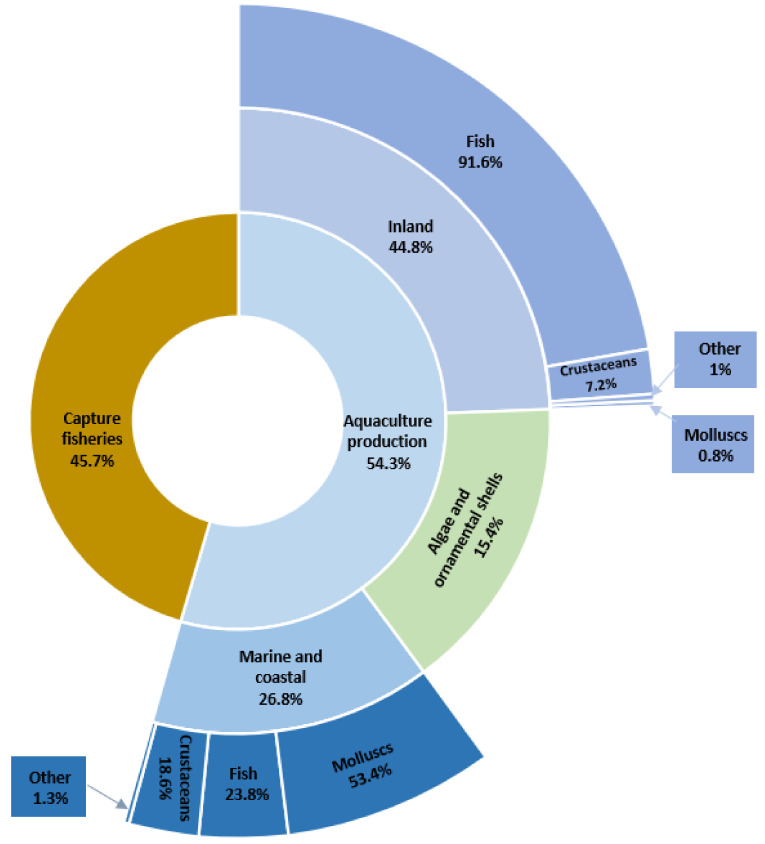
Percentage of global catches and aquaculture in 2018 (based on [1]).

Agriculture, along with aquaculture, provides most of the food that the world population needs. Fish and aquaculture products are recognized not only as some of the healthiest foods on the planet but also as some of the least harmful to the environment [1]. According to estimates by the Food and Agriculture Organization of the United Nations (FAO), aquaculture is one of the fastest-growing food production sectors in the world. The latest statistics show that the total world production of aquatic organisms reached a record of 210.9 million tonnes in live weight in 2018. Aquaculture accounted for 54.3% of this production and consisted of 82.1 million tonnes of aquatic animals and 32.6 million tonnes of algae, ornamental shells, and pearls. The dominant segment was fish production (Figure 1). The total number of FAO-registered farm aquatic animal species in 2018 was 622. Sales of global aquaculture production excluding algae and ornamental shell production are estimated at $250 billion [1].

The rapid increase in aquaculture production raises concerns related to the quality and safety of aquatic-animal health. As in other livestock-production sectors, aquatic farming also uses intensive and semi-intensive practices, leading to a greater density of animals in small water spaces and significantly increasing the risk of developing infectious diseases [2]. Bacterial diseases affecting crops, fish, and crustaceans not only cause large economic losses to producers but can even cause food shortages, resulting in malnutrition in vulnerable populations [3].

Bacterial diseases which are routinely encountered in aquaculture and contribute to production failure or decline are mainly caused by Gram-negative bacteria such as *Aeromonas* (*A.*) *hydrophila*, *A. salmonicida*, *Edwardsiella tarda*, *Flavobacterium psychrophilum*, *Pseudomonas fluorescens*, and various *Vibrio (V.*) species. Much less often, diseases are caused by Gram-positive bacteria, such as *Streptococcus iniae*, *Renibacterium salmoninarum*, or *Mycobacterium* sp. [4,5,6]. Most of the bacterial pathogens that cause problems in aquaculture occur naturally in the aquatic environment, both freshwater and marine. External stressors, including transport, high stocking densities, poor water quality, and inadequate nutrition can predispose animals to disease.

The actual amount of antimicrobials used in food-producing animals is difficult to estimate due to incomplete information, but 2018 sales data for 31 EU member states indicate that 6400 tons of antimicrobials were used for veterinary purposes, primarily for food-producing animals [7]. In the United States, domestic sales and the distribution of medically important antimicrobial drugs approved for use in food-producing animals totaled 11,500 tons in 2019 [8]. This is a particularly important problem because almost all antimicrobial agents used in animal husbandry are similar in structure or are identical to those used in human medicine, which promotes the formation of multi-drug resistant strains and cross-resistance. 

**Figure 2:**
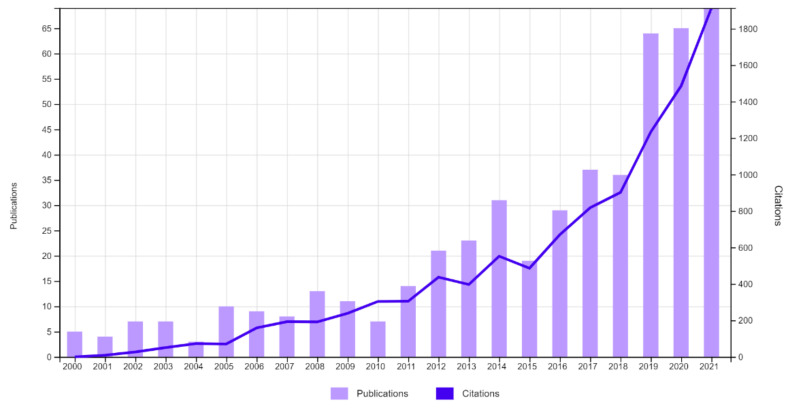
The number of records for the words “phage” and “aquaculture” in the years 2000–2021 in the Web of Science All Database.

As we approach what could be a “post-antibiotic era” as announced by the WHO, there is growing interest in alternative tools that will reduce the use of antibiotics. One of the eco-friendly alternatives considered and a possible solution to the antimicrobial resistance crisis is the use of bacteriophages. The use of bacteriophages is not a new concept, and the results of its use have been described in human medicine, veterinary medicine, agriculture, and the food industry, but its use in aquatic animal husbandry has recently gained more interest. An analysis of the number of records containing the words “phage” and “aquaculture” in the Web of Science All Database from 2000 to 2021 reveals a rapid increase in the number of scientific reports related to the subject of bacteriophages in combination with the aquatic environment, which suggests a significant increase in interest in the need to research the subject (Figure 2).

## 2. Antibiotic Resistance in Aquaculture

Antimicrobials in aquaculture are used for both prophylactic and therapeutic purposes and are usually administered to the entire population of sick, healthy, and vector animals through a process known as metaphylaxis. For this reason, the amount of antibiotics used in aquaculture is proportionally higher than that used in land-animal farming. Antimicrobial agents are administered to aquaculture animals mainly in feed, rarely by injection or immersion. Of the ingested antimicrobials, about 80% pass into the environment as unabsorbed in feces or, after absorption, in urine and other secretions. Additionally, if the fish are sick and anorexic, uneaten food containing drugs (possibly as much as 30%) is gravitationally deposited into sediments from which they can be flushed away by currents and carried to distant locations. Residual antibiotics remain in the sediment, thus changing the composition of the sediment microbiota, allowing for the development of antibiotic-resistant bacteria [9,10]. In surface waters, it is difficult to find an area where no antibiotic residues are detected. The exceptions are places near the source, where rivers or streams have not yet passed through urban or agricultural areas [11]. Some antibiotics can even be found in groundwater below 10 m [12].

The classes and amounts of antibiotics used in agriculture and aquaculture depend on the region of the world studied. Between 2008 and 2018, 67 different antibiotics were used in 11 of the world’s leading aquaculture-producing countries, with the main users being Vietnam (39), China (33), and Bangladesh (21) [13]. Compared to a report published 13 years ago [14], when an average of seven antibiotics were used between 1990 and 2007, a drastic increase has occurred, possibly due to the widespread prophylactic use of drugs in Vietnam and China. The lists of banned antibiotics in these countries were recently updated [15,16], but these products are still detected in aquaculture products. 

The extensive and frequent use of antibiotics in aquaculture in the past has resulted in the development of pathogen resistance, representing one of the main challenges in aquaculture. The first recorded pathogen of fish showing resistance to antimicrobial agents (against sulfathiazole and tetracycline) was *A. salmonicida* [17]. Currently, such pathogens are isolated from many farmed fish and crustaceans around the world [18,19] (Table 1).

The WHO report on the global surveillance of resistance states indicates that existing antimicrobials are becoming less effective [40,41]. At the same time, there is limited work focused on developing new ones. Although preventive vaccines are available against many bacterial infections, their use is still limited. If this trend continues, tools to combat resistant microorganisms will soon be exhausted [40]. The choice of effective therapeutic methods is therefore problematic at present. There exists a need to develop ways to protect aquaculture animals from pathogenic bacteria without the use of antibiotics. A holistic approach that considers the relationship between pathogen, host, and environment seems necessary in the long term.

## 3. Bacteriophages

Bacteriophages (bacterial viruses, phages) are the most abundant microorganisms on Earth. They occur almost everywhere, including in extreme environments [42], as well as in almost all niches of human and animal organisms [43]. They were independently discovered by Frederick W. Twort in England in 1915 and by Felix d’Herelle at the Pasture Institute in Paris in 1917 [44,45]. These are viruses that infect and multiply in bacteria and archaea. They are extremely varied in size, morphology, and genome organization, however, almost all the currently classified bacteriophages are assigned to only three families of the caudate bacteriophage, which is assigned to the order *Caudovirales* [46]. The differences between the three families are as follows: a long or short shrink tail (*Myoviridae*), a long non-shrink tail (*Siphoviridae*), and a short non-shrink tail (*Podoviridae*) [47].

### 3.1. The Life Cycle of Bacteriophages

Bacteriophages, similarly to other viruses, must infect the host cell to reproduce. They are very specific to their hosts and usually infect only one species of bacteria or even certain strains within a species. Bacteriophages can recognize several components of the bacterial cell structure as their receptors. These include, inter alia, outer membrane proteins, peptidoglycan (PG), teichoic acids, oligosaccharide, lipopolysaccharide (LPS), flagella, and fimbriae [48]. This means that they can only infect bacteria that have a target molecule to bind to [42,49].

The first step in a tightly programmed bacteriophage infection process is the production of polysaccharide-degrading enzymes, also known as polysaccharide depolymerases. These can be released into the environment, associated with the tail or capsid of the bacteriophage, and are used for the enzymatic degradation of the envelope or structural polysaccharides, including exopolysaccharides which are the main component of the bacterial biofilm. During the last phase of the cycle, lysines are produced, which are responsible for the lysis of bacteria and the release of progeny viruses [48,50].

Lytic and lysogenic infections are most frequently mentioned in the literature. However, these are only two of the many possibilities. Not all infections necessarily result in the death of the host cell, and the replication of bacteriophage particles does not always occur. Each bacteriophage can follow several different infection pathways depending on the environmental conditions and the genetic and physiological characteristics of the host.

#### 3.1.1. Lytic Cycle 

While they are technically not living organisms, bacteriophages are certainly dynamic entities. During the lytic replication cycle, the bacteriophage attaches to a sensitive host cell. It then introduces its genome into the bacterial cytoplasm and uses its ribosomes to produce proteins. Bacterial resources are rapidly transformed in the capsid protein and the virus genome, which consists of multiple copies of the original bacteriophage. By multiplying in a bacterial cell, it destroys it. When the host cell dies, it most often releases new bacteriophages with the participation of lysines, which then infect another bacterial cell [51,52]. Such phages are termed virulent or lytic.

#### 3.1.2. Lysogenic Cycle

In the lysogenic replication cycle, the bacteriophage also attaches to a susceptible bacterial cell and introduces its genome into the bacterial cytoplasm. The bacteriophage genome is then integrated into the chromosome of the bacterial cell or remains unbound. In both cases, it is replicated and transferred to progeny bacterial cells without lysing them. The integrated bacteriophage genomes are called prophages. Prophages can return to the lytic replication cycle leading to the lysis of their host, most often in response to changing environmental conditions [51,52,53]. 

#### 3.1.3. Pseudolysogenic Cycle

During pseudolysogeny, the bacteriophage enters the cell, but does not replicate in the cell and does not integrate stably with the host genome. It seems that pseudolysogeny plays an important role in the survival of bacterial viruses, enabling the preservation of its genome when the host cell encounters unfavorable growth conditions, such as the lack of a sufficient amount of nutrients [54]. Pseudolysogeny is not a permanent state. After changing the conditions causing it, the bacteriophage often enters the lytic or lysogenic pathway. Sometimes the term “carrier” is used instead of the term pseudolysogeny [42,51].

#### 3.1.4. Chronic Infection

In the case of chronic infection, new bacteriophage particles are produced continuously over a long period. However, lysis of the host cell does not occur. Virions are released or are exported out of the cell by protein complexes. It is associated with high energy expenditure and may negatively affect the ability of bacteria to compete for an ecological niche. Examples of bacteriophages capable of causing a chronic infection are some archaeal viruses, filamentous phages (ssDNA phages), and mycoplasma-infecting plasmaviruses [42].

#### 3.1.5. Abortive Infection

Faced with frequent exposure to bacteriophages, bacteria have developed numerous mechanisms to counteract infection, including abortion infection, also called bacteriophage exclusion. The bacteriophage genome enters the host cell, but the infected cell self-destructs before the bacteriophage completes its replication cycle. This reduces the number of progeny particles and limits their spread to other cells, allowing the bacterial population to survive [55]. The abortive infection manifests itself in a wide variety of bacterial defense systems. An example is the toxin/antitoxin system of the genus *Lactococcus*. In uninfected bacteria, the action of both components of the system is balanced. After infection by the virus, bacterial death occurs due to an increase in the toxin–antitoxin ratio [55,56].

### 3.2. Bacteriophage Therapy

For targeted therapy, strictly lytic bacteriophages are preferred, characterized by rapid multiplication leading to the lysis of the bacterial cell, and at the same time by an exponential increase in their number. Lysogenic bacteriophages are avoided because of their inherent ability to mediate gene transfer between bacteria, which can increase bacterial virulence, for example by promoting antibiotic resistance. Currently, advances in sequencing technologies and synthetic biology are creating new possibilities for using these bacteriophages in the treatment of bacterial infections as well. In addition to the ability to lyse a bacterial cell, several important features determine the antimicrobial efficacy of bacteriophages. One is the bacteriophage generation time, which includes effective adhesion, the latent period, and the release of progeny particles. The second aspect is the growth rate of the bacteriophage population, which is the number of bacteriophage particles formed during one life cycle. High adsorption rate to specific bacteria, large burst size, and short generation time are determinants of strong antibacterial efficacy [48].

#### 3.2.1. Methods of Administering Bacteriophages

The effectiveness of the application of bacteriophages depends on their ability to reach the host, which is not always possible. Depending on the location of the infection in the body, and due to the effectiveness of penetration and the ability to maintain the highest bacteriophage titer, preparations containing bacteriophages can be administered in various forms. Bacteriophages used to combat bacterial diseases in aquaculture can be administered orally with feed [57,58,59,60], parenterally (intramuscularly, subcutaneously, intraperitoneal) [58,59,61,62,63], topically to the skin and lesions [64], in a bath [60,65,66] or can be directly released in the water system [67,68,69,70].

In aquaculture, methods that reduce the need to perform additional activities are preferred, and thus limit exposure to manipulation stress; therefore, the most frequently chosen method of administering drugs is in water or orally, e.g., with feed. The oral administration of bacteriophages has been proven to be effective in treating gastrointestinal infections. The absorption of orally administered bacteriophages into the systemic circulation in a process similar to bacterial translocation has also been demonstrated, which allows this route of administration to also be used in systemic infections. The passage of bacteriophages is determined by several factors, including their concentration, the presence of specific sequences within the capsid proteins that interact with enterocyte receptors, and the interaction of bacteriophages with intestinal immune cells [71]. Application in water is the most common method of applying different substances to aquaculture animals. It is used in several ways, from high drug concentration/short exposure time (bath) to low drug concentration/long exposure time (immersion) [72]. The application of bacteriophage preparations with these methods is very popular, not only because of the ease of administration resulting in bacteriophages entering internal organs directly from the water through the gills of fish, but due to the additional benefits of cleaning the environment [73]. However, the use of this route of administration can be problematic in commercial-scale aquaculture, where the volume of water requiring bacteriophage treatment can be impractically large.

Unfortunately, there is no universal application. The appropriate method of bacteriophage administration depends on many factors and situations should be considered on a case-by-case basis. It is not practical to perform injections on very small fish or crustaceans. Similarly, performing bathing with a high bacteriophage titer is difficult in large bodies of water, and the immersion method may depend on the environment, the nature of the infection, or the bacteriophage properties [74]. Each method of administration has its strengths and weaknesses, and the choice of method largely depends on the nature of the bacterial pathogen, the species of animal, and its size. 

There are two forms of therapy—active and passive. In active therapy, bacteriophages are administered at a dose that is capable of reducing the host population through multiple cycles of reproduction. In passive therapy, such reproduction is not needed to ensure an effective therapy since the number of bacteriophages administered is so large that the entire host population is lysed without the need for one or more cycles of bacteriophage reproduction. Unfortunately, the passive method is much more expensive but can bypass bacterial defense mechanisms such as abortive infection [73,75].

Various approaches to bacteriophage therapy have been tested. Monophage therapy refers to the use of one type of bacteriophage. It is used primarily for the development of experimental models of bacteriophage therapy, as a confirmation of the concept when testing preparations. Unfortunately, it requires the precise matching of the pathogen and the bacteriophage.

In aquaculture, the actual situation is often much more complicated than “one pathogen-one disease”. Fish can suffer simultaneously from infections caused by multiple strains or species of bacteria that can affect the outcome of the disease which creates additional challenges for phage therapy [76]. Polyphage therapy with a bacteriophage cocktail uses a combination of several phages. Unlike monophage therapy, it targets many strains of one bacterial species or many bacterial species. The use of bacteriophage cocktails containing two or more bacteriophages is increasingly being tested in aquaculture. One of the benefits of using multiple bacteriophages is that they allow for a more thorough treatment of infection as they can attack a wide range of pathogenic bacterial strains, with better bacterial titer reduction and faster-acting effects [77]. In addition, the use of bacteriophage cocktails targeting different receptors of the same bacterium may help reduce the rate of resistance development [73].

#### 3.2.2. In Vivo Use of Bacteriophages

In the human and animal health sectors, bacteriophage therapy has been practiced in regions of Eastern Europe for over 60 years [78]. Between 1930 and 1940, the discovery of antibiotics led to the abandonment of bacteriophage therapy in Western countries; meanwhile, due to the isolation of many Eastern European countries from the advances in the production of antibiotics, the region continued to develop and improve bacteriophage therapies at that time. 

The first use of bacteriophages as a therapy in aquaculture was described by Wu et al. in 1981 [79]. Since then, interest in bacteriophage therapy in various species of aquatic animals has attracted a lot of attention, including in the control of diseases caused by *Aeromonas* spp., *Pseudomonas* spp., *Yersinia ruckeri*, *Flavobacterium psychrophilum*, and many others. In recent years, several in vivo experiments have been performed to assess the potential of bacteriophages to combat bacterial infections in aquaculture. Their effectiveness was tested on various animal models, including many species of fish, crustaceans, and mollusks, showing promising results and revealing the ability of certain bacteriophages to significantly reduce pathogen concentrations and increase the survival rate of aquaculture animals. The main achievements of in vivo studies of bacteriophages specific for pathogenic bacteria in aquaculture animals and their potential uses are presented in Table 2.

Most of the published articles discuss monophage therapy and describe the discovery of new potentially useful bacteriophages, while few describe cocktails or other associations. The presented in vivo experiments demonstrate the effectiveness of bacteriophage therapy in controlling aquaculture-related diseases; however, the results for different combinations of bacteriophages and bacteria varied. They range from 100% bacterial removal and lack of mortality [57,91] to no therapeutic effect [60]. In some cases, disease progression was delayed; however, the final mortality did not differ statistically from the group where bacteriophage therapy was not used [90,103].

The ratio of bacteriophage to bacterial concentration required for effective active bacteriophage therapy varies widely, depending on the pathogen, fish species, and bacteriophage. Different doses were used in both the experimental and field studies. Le et al. (2018) [83], using different amounts of bacteriophage in catfish therapy, obtained survival rates that varied by up to 68%, indicating that determining the correct bacteriophage dose is very important for successful bacteriophage therapy.

The time of administration was also found to be a very important factor. Mortality typically increased significantly when treatment was delayed from time of infection [91,97,99]. The best choice seems to be the prophylactic administration of bacteriophages before infection and its continuation. Jun et al. (2018) [121] achieved a 75% higher survival rate for white shrimp with the prophylactic use of bacteriophages in immersion, and a 50% survival rate with prophylaxis in feed. After the administration of the bacteriophage preparation a day before the infection, Schulz et al. [86,87] achieved a 16% higher survival of European eel and 6% higher survival of rainbow trout, compared to the group treated 24 h after infection. This may be related to the time needed for bacteriophages to multiply to a sufficient concentration to cause the host population to collapse. Further research into the effect of timing and bacteriophage number on the success of bacteriophage therapy may yield interesting results, as a more concentrated administration may compensate for delayed treatment [73]. 

The most important aspect is to compare the effectiveness of therapy with bacteriophages to that of antibiotic therapy. Zhang et al. [106] found no statistical differences in the survival rate of the sea cucumber infected with *V. alginolyticus* after treatment with a bacteriophage cocktail compared to the group treated with antibiotics. Karunasagar and co-authors [113] achieved a 20% higher survival rate of shrimps treated with bacteriophages compared to survival with the use of antibiotics, while Vinod et al. [114] achieved 46% higher survival compared to antibiotic treatment after natural *V. harveyi* infection of shrimp. This suggests that the effectiveness of bacteriophage therapy may not only match that of antibiotics but may even be more effective. However, further studies are needed to both compare the efficacy of antibiotics and bacteriophages, and to study the extent and rate of bacteriophage resistance.

## 4. Conclusions

The production of various species of fish, crustaceans, and mollusks has made the aquaculture industry an important economic factor in many countries. Despite advances in good management practices, non-specific immunoprophylaxis, and vaccine production, bacterial infections remain a serious problem both in hatcheries and during rearing, often resulting in a high level of mortality. An additional problem is that more and more of them have been characterized by multi-drug resistance [47]. In the absence of an adequate strategy to combat bacterial pathogens, alternative, environmentally friendly disease control strategies should be developed that should reduce the risk of the development and spread of microbial resistance. In line with this idea, the use of bacteriophage therapy in aquaculture seems very promising.

Therapy with bacteriophages has a few advantages over traditional antibiotic therapy. Phage isolation is relatively rapid, simple, and inexpensive. Bacteriophage resistance develops about ten times slower than antibiotic resistance because bacteriophages can evolve, creating new genotypes capable of re-infecting a given bacterial strain [124]. Bacteriophages remain infectious under very harsh environmental conditions and tend to continue to replicate until the host bacterial population density is significantly reduced. These features indicate that bacteriophage therapy—unlike traditional therapies—may require fewer administrations and at the same time work as well or better than conventional treatments [125].

Several key issues must be considered in practical application to achieve the desired outcome, reduction, or elimination of mortality due to bacterial infection. First, careful planning of the timing and frequency of bacteriophage administration should be performed, keeping in mind the virulence characteristics of the bacterial pathogen. Second, the optimal route of bacteriophage administration for each bacterial infection should be determined, considering that pathogenic bacteria have different routes of infection. Third, the appropriate bacteriophage dose should be determined, which will depend on the expected number of target bacteria [76]. Some very pertinent issues have yet to be addressed before the widespread use of bacteriophage treatment is made possible, including the presence of bacteriophage-resistant bacteria, the high specificity of bacteriophages and the transfer of virulence genes. In some cases, the reproduction of the lytic bacteriophage leads to undesirable consequences, which should also be considered. The rapid release of cellular toxins or the breakdown of the outer membrane of Gram-negative bacteria in the short term can result in a systemic inflammatory response and severe side effects [126]. Bacteriophage treatment also requires a consideration of factors such as cost-effectiveness, environmental impact, and more importantly, it must be standardized and specified to geographic regions and species [74].

Most studies with pathogens from aquaculture have been conducted under controlled laboratory conditions. Future studies conducting experiments in conditions resembling the real-life rearing environment are important to develop a better understanding of the efficiency of bacteriophage treatments. It is also necessary to promote knowledge about bacteriophages for consumer acceptance of bacteriophage-based products. Given the renewed interest and enthusiasm in the field of bacteriophage therapy, there is reason to believe that these challenges can be overcome in the years to come.

## Figures and Tables

**Table 1:** Examples of antimicrobial-resistant aquaculture pathogens.

Pathogen	Species	Location	Ineffective Antimicrobials	References
*Aeromonas aquariorum*	Pacific whiteleg shrimp (*Litopenaeus vannamei*), Tiger prawn (*Penaeus monodon*)	Thailand	ampicillin, ampicillin + sulbactam, cephalothin, cefotaxime, erythromycin, tetracycline, clindamycin, nalidixic acid, norfloxacin, trimethoprim-sulfamethoxazole	[20]
*Aeromonas hydrophila*	Channel catfish (*Ictalurus punctatus*)	United States	ampicillin, chloramphenicol, kanamycin, nitrofurantoin, oxytetracycline, tetracycline	[21]
*Aeromonas* spp.	Goldfish (*Carassius auratus*)	United States	ampicillin, furadantoin, sulfadiazine, sulfadimethoxine + ormetoprim, tetracycline, others	[22]
	Rainbow trout (*Oncorhynchus mykiss*)	Denmark	amoxicillin, oxolinic acid, oxytetracycline, sulfadiazine + trimethoprim,	[23]
	Rainbow trout (*Oncorhynchus mykiss*)	Australia	amoxicillin, cephalothin, ceftiofur, chloramphenicol, florfenicol, nitrofurantoin, streptomycin, sulfamethoxazole, tetracycline, ticarcillin, trimethoprim	[24]
	Rainbow trout (*Onchorynchus mykiss*)	Mexico	β-lactams	[25]
	Ornamental fish	India	amoxicillin, cephalothin, cefpodoxime, carbenicillin, nalidixic acid, streptomycin, tetracycline, trimethoprim	[26]
	Mozambique tilapia (*Oreochromis mossambicus*), Rainbow trout (*Oncorhynchus mykiss*), Carp (*Cyprinus carpio*)	South Africa	ciprofloxacin, nalidixic acid, ofloxacin	[27]
*Aeromonas sobria*	Koi carp *(Cyprinus carpio koi)*	Czech Republic	quinolones, sulfonamides, tetracycline	[28]
*Aeromonas veronii*	Pacific whiteleg shrimp (*Litopenaeus vannamei*), Tiger prawn (*Penaeus monodon*)	Thailand	ampicillin, ampicillin + sulbactam, cephalothin, erythromycin, imipenem, clindamycin, nalidixic acid, norfloxacin, tetracycline, trimethoprim + sulfamethoxazole	[20]
*Edwardsiella tarda*	Olive flounder (*Paralichthys olivaceus*)	South Korea	kanamycin, streptomycin, tetracycline	[29]
	Turbot (*Scophthalmus maximus)*	China	chloramphenicol	[30]
*Escherichia coli*	Gilt-head bream (*Sparus aurata*)	Portugal	β-lactams	[31]
*Enterobacteriacae*	Carp (*Cyprinus carpio*), Rainbow trout (*Salmo gairdneri*), Bighead carp (*Hypophthalmichthys nobilis*)	Lithuania	ampicillin, β-lactams, second-generation cephalosporins, carbapenems	[32]
*Flavobacterium psychrophilum*	Rainbow trout (*Oncorhynchus mykiss*)	Denmark	amoxicillin, oxolinic acid, oxytetracycline, sulfadiazine + trimethoprim	[23]
	Rainbow trout (*Oncorhynchus mykiss*), Atlantic salmon (*Salmo salar*), Trout (*Salmo trutta*)	Norway	quinolones	[33]
*Plesiomonas shigelloides*	Channel catfish (*Ictalurus punctatus*)	United States	ampicillin, chloramphenicol, kanamycin, nitrofurantoin, oxytetracycline, tetracycline	[21]
*Photobacterium damselae*	Yellowtail (Seriola quinqueradiata)	Japan	chloramphenicol, kanamycin, sulfonamide, tetracycline	[34]
	Palmetto bas (*Morone saxatilis* × *M. chrysops)*	United States		
*Pseudomonas aeruginosa*	Gilt-head bream (*Sparus aurata*)	Tunisia	ampicillin, chloramphenicol, erythromycin, tetracycline	[35]
*Pseudomonas* spp.	Carp (*Cyprinus carpio*), Rainbow trout (*Salmo gairdneri*), Bighead carp (*Hypophthalmichthys nobilis*)	Lithuania	β-lactams	[32]
	Rainbow trout (*Oncorhynchus mykiss*)	Australia	amoxicillin, cephalothin, ceftiofur, ticarcillin, chloramphenicol, florfenicol, streptomycin, nitrofurantoin, and trimethoprim	[24]
*Streptococcus dysgalactiae*	Mullet (*Mugil cephalus*), Cobia (*Rachycentron canadum),* Golden pompano (*Trachinotus blochii),* Amberjack (*Seriola dumerili*), Yellowtail (*Seriola quinqueradiata),* others	Taiwan and Japan	erythromycin and tetracycline	[36]
*Vibrio harveyi*	*Penaeidae*	India	ampicillin, ceprofloxacin, chlortetracycline, erythromycin, furazolidone, gentamicin, nalidixic acid, neomycin, novobiocin, oxytetracycline, penicillin G, polymyxin B, rifampicin, streptomycin	[37]
	Tiger shrimp (*Penaeus monodon*)	Philippines	chloramphenicol, furazolidone, oxolinic acid, oxytetracycline	[38]
*Vibrio* sp.	Yellowtail (*Seriola quinqueradiata*)	Japan	oxytetracycline	[39]
*Yersinia ruckeri*	Rainbow trout (*Oncorhynchus mykiss*)	Denmark	oxolinic acid	[23]

**Table 2:** Outcomes of in vivo bacteriophage application in aquaculture.

Pathogen	Species	Application	Outcome	References
*Aeromonas hydrophila*	Carp (*Cyprinus carpio*)	Intraperitoneal injection	Reduction in mortality by 100%, 60% or 50% depending on the bacteriophage or cocktail used.	[80]
	Cyprinid loach (*Misgurnus anguillicaudatus*)		1. Mortality drop from 39% to 0%; 2. A decrease in mortality from 100% to 43% or 17% depending on the used bacteriophage.	[58]
			No mortality after 7 days compared to control group (65%)	[79]
		Feed	1. A decrease in mortality from 39% to 17% or 11% depending on the used bacteriophage; 2. A decrease in mortality from 96% to 47% or 27% depending on the used bacteriophage.	[58]
		Bath	A 47% decrease in mortality; most surviving fish showed no signs of disease.	[66]
	Nile tilapia (*Oreochromis niloticus*)	Intraperitoneal injection	A 50% decrease in mortality.	[81]
		Immersion	Reduction in mortality by 37.5–55% depending on bacteriophage dose.	[82]
	Rainbow trout (*Oncorhynchus mykiss*)	Intraperitoneal injection	Reduction in mortality by 40% after prophylactic administration.	[59]
		Feed	Reduction in mortality by 70% after prophylactic administration.	
		Bath	Reduction in mortality by 80% after prophylactic administration.	
	Striped Catfish (*Pangasianodon hypophthalmus*)	Intraperitoneal injection	Reduction in mortality by 82%, 37% or 14% depending on bacteriophage dose.	[83]
		Feed	Reduction in mortality by 51.6–60% depending on bacteriophage dose.	[84]
	Zebrafish (*Danio rerio*)	Immersion	Reduction of mortality by 43.3%.	[85]
*Aeromonas hydrophila* and *Pseudomonas fluorescens*	European eel (*Anguilla anguilla*)	Bath	Reduction in mortality by 40%, 25% or 15% depending on time of initiation of therapy; reduction in mortality by 60% with prophylactic use.	[86]
	Rainbow trout (*Oncorhynchus mykiss*)	Bath	Reduction in mortality by 25%, 15% or 10% depending on time of initiation of therapy; reduction in mortality by 36% with prophylactic use.	[87]
*Aeromonas salmonicida*	Brook trout (*Salvelinus fontinalis*)	Immersion	Delayed disease onset by 7 days and reduced mortality from 100% to 10%	[88]
	Rainbow trout (*Oncorhynchus mykiss*)	Intramuscular injection	Reduction in mortality from 100% to 70%.	[61]
	Senegalese sole (*Solea senegalensis*)	Immersion	No mortality compared to the control group (36%).	[89]
*Aeromonas salmonicida* subsp. *salmonicida*	Atlantic salmon (*Salmo salar*)	Intraperitoneal injection	Delayed mortality; final mortality did not differ between groups.	[90]
		Feed		
		Bath		
	Rainbow trout (*Oncorhynchus mykiss*)	Intramuscular injection	Reduction in mortality by 26.7%, no symptoms up to 14 days after bacteriophage administration.	[61]
*Citrobacter freundii*	Carp (*Cyprinus carpio*)	Intraperitoneal injection	Reduction in mortality by 100%, 45% and 0% depending on time of bacteriophage administration.	[91]
*Citrobacter* spp.	Zebrafish (*Danio rerio)*	Bath	Reduction in mortality by 17%, 23% and 26% depending on the bacteriophage or cocktail used	[92]
*Edwardsiella tarda*	Turbot (*Scophthalmus maximus*)	Feed	Reduction in mortality by 53%, 76% or 80% depending on bacteriophage dose.	[93]
	Zebrafish (*Danio rerio)*	Bath	Reduction in mortality by 50%.	[94]
*Flavobacterium columnare*	Rainbow trout (*Oncorhynchus mykiss*)	Bath	Reduction of mortality by 33–42% depending on the number of bacteriophages.	[65]
	Walking catfish *(Clarias batrachu*)	Intramuscular injection	No symptoms and 100% survival.	[57]
		Bath		
		Feed		
	Zebrafish (*Danio rerio*)	Immersion	Reduction in mortality by 60%.	[65]
*Flavobacterium psychrophilum*	Atlantic salmon (*Salmo salar*)	Intraperitoneal injection	Mortality decreased from 45% to 18% and from 13% to 6% depending on the bacteriophage used.	[95]
	Rainbow trout (*Oncorhynchus mykiss*)		Mortality decreased from 47% to 20% and from 80% to 47% depending on the bacteriophage used.	
			23% reduction in mortality by phage administration 3 days after infection.	[60]
			Cocktail reduced mortality by 17–54% depending on the bacteriophage/bacterial ratio.	[96]
		Feed	No significant differences in final mortality.	[60]
		Bath		
*Lactococcus garvieae*	Japanese amberjack (*Seriola quinqueradiata*)	Intraperitoneal injection	Mortality decreased from 90% to 0–50% depending on the timing of bacteriophage administration.	[97]
		Feed	Mortality reduced from 65% to 10%.	
	Rainbow trout (*Oncorhynchus mykiss*)		Reduction in mortality from 100% to 70% after 2 weeks.	[98]
*Photobacterium damselae subsp*. *Damselae*	Longfin yellowtail (*Seriola rivoliana*)	Immersion	Increased egg hatch rate from 50% to 80%.	[70]
*Pseudomonas aeruginosa*	African catfish (*Clarias gariepinus*)	Locally on skin lesions	A seven-fold reduction in the size of the lesions.	[64]
*Pseudomonas plecoglossicida*	Aju sweetfish (*Plecoglossus altivelis*)	Feed	Reduction of mortality by 42.5% when bacteriophages were administered at the time of infection in 10 g fish; in 2.4 g fish by 78% and 67% depending on time of administration.	[99]
			1. Reduction in mortality by 40% and 73% with the cocktail; 2. Mortality reduced by 73% and 63% depending on the bacteriophage used; 3. Field infection- reduction in mortality from 18 kg per day to 6 kg after 3 applications of bacteriophage.	[100]
*Streptococcus agalactiae*	Nile tilapia (*Oreochromis niloticus*)	Intraperitoneal injection	A 3-day delay and 40% reduction in mortality.	[63]
*Streptococcus iniae*	Japanese flounder (*Paralichthys olivaceus*)	Intraperitoneal injection	The decrease in mortality by 28–90% depending on the dose and time of administration.	[101]
*Streptococcus parauberis*	Japanese flounder (*Paralichthys olivaceus*)	Feed	Improved fish growth, reduced bacterial detection and improved breeding survival.	[102]
*Vibrio alginolyticus*	Atlantic cod (*Gadus morhua*)	Immersion	Mortality delay; no statistically significant differences at the end of the experiment.	[103]
	Atlantic salmon (*Salmo salar*)		1. Reduction in mortality from 93% to 0–30% depending on the dose of bacteriophages under experimental conditions; 2. Reduction in mortality from 40% to 0% in breeding conditions.	[104]
	*Artemia salina*		The total load of bacteria decreased by 93%.	[105]
	Japanese sea cucumber (*Apostichopus japonicus*)	Feed	70%, 47% and 44% reduction in mortality after using a cocktail depending on the dose; no difference in survival compared to the use of antibiotics.	[106]
	New Zealand rock oyster (*Saccostrea glomerata*)	Immersion	Reduction of larvae mortality by 50% after using a cocktail.	[69]
	Turbot (*Scophthalmus maximus*)		Mortality delay; no statistically significant differences at the end of the experiment.	[103]
*Vibrio anguillarum*	Zebrafish (*Danio rerio*)	Immersion	Mortality reduced from 17% to 3%.	[107]
*Vibrio campbellii*	*Artemia franciscana*	Immersion	Survival of nauplii increased by 24%.	[108]
*Vibrio coralliilyticus*	Pacyfic oyster (*Crassostrea gigas*)	Immersion	Reduction in larvae mortality after prophylactic use.	[109]
*Vibro cyclitrophicus*	Japanese sea cucumber (*Apostichopus japonicus*)	Feed	Mortality reduced from 81% to 18%.	[62]
		Injection into the body cavity	Mortality reduced from 58% to 18%.	
		Immersion	Mortality reduced from 63% to 18%.	
*Vibrio harveyi*	Brine shrimp (*Artemia franciscana*)	Immersion	Bacteriophage cocktails enhanced hatching success (100%, control groups had a hatching success of around 50%) and survival rate (85–89%, control groups survival rate was 40–50%).	[110]
	*Artemia salina*		Larval mortality decreased 24 h post-infection.	[111]
	Giant tiger prawn (*Penaeus monodon*)	Immersion	Larval mortality decreased by ∽43%.	[112]
			Reduction in mortality of larvae by 20% compared with antibiotic therapy.	[113]
			1. In experimental infection, larvae mortality decreased by 55%; 2. With a natural outbreak, larvae mortality decreased by 69% compared to the untreated control, by 46% compared to the antibiotic treatment group.	[114]
			Reduction of larvae mortality by 50%.	[115]
	Greenlip abalone (*Haliotis laevigata*)	Bath	Reduction in mortality of 70% compared with the control group.	[116]
	Turbot (*Scophthalmus maximus*)	Feed	Reduction in mortality by 58–28% depending on bacteriophage dose.	[117]
	Zebrafish (*Danio rerio*)	Intraperitoneal injection	1. Reduction in mortality by 27.7–33.3% depending on infectious dose with prophylactic bacteriophage application; 2. Reduction in mortality by 13.3–26.7% depending on infectious dose with therapeutic bacteriophage application.	[118]
*Vibrio parahaemolyticus*	*Artemia franciscana*	Immersion	Increase in breeding success and larval survival both when using a single bacteriophage and a cocktail.	[110]
			Depending on the bacterial strain, larval mortality decreased by 35% or to a level comparable to the uninfected control.	[68]
	Blue mussel (*Mytilus edulus*)	Immersion	Reduction of the bacteria number to undetectable levels in the tissues.	[67]
	Giant tiger prawn (*Penaeus monodon)*	Feed	Reduction in mortality of 40–45% by single bacteriophage and 50% by a cocktail.	[119]
	Whiteleg shrimp (*Litopenaeus vannamei*)	Immersion	Reduction in mortality of larvae in 18–21% depending on the bacteriophage; delayed therapy resulted in decreased larval survival.	[120]
			Reduction in mortality of 75 and 50% depending on time after prophylactic use; no effect of therapeutic use.	[121]
		Feed	Reduction in mortality of 50% for prophylactic use, no effect of therapeutic use.	
			20–40% dose-dependent reduction in mortality.	[122]
*Vibrio splendidus*	Japanese sea cucumber (*Apostichopus japonicus*)	Feed	Reduction in mortality of 32–47% by a single bacteriophage, and 64% by a cocktail.	[123]

## Data Availability

Not applicable.
